# Smartphone Location-Independent Physical Activity Recognition Based on Transportation Natural Vibration Analysis

**DOI:** 10.3390/s17040931

**Published:** 2017-04-23

**Authors:** Taeho Hur, Jaehun Bang, Dohyeong Kim, Oresti Banos, Sungyoung Lee

**Affiliations:** 1 Department of Computer Science and Engineering, Kyung Hee University, (Global Campus), 1732, Deogyeong-daero, Giheung-gu, Yongin-si, Gyeonggi-do 17104, Korea; hth@oslab.khu.ac.kr (T.H.); jhb@oslab.khu.ac.kr (J.B.); dhkim@oslab.khu.ac.kr (D.K.);; 2 Telemedicine Cluster of the Biomedical Signals and Systems Group, University of Twente, Enschede 7500AE, The Netherlands; o.banoslegran@utwente.nl

**Keywords:** movement activity recognition, vehicle natural vibration, natural vibration feature extraction, correction algorithm, smartphone

## Abstract

Activity recognition through smartphones has been proposed for a variety of applications. The orientation of the smartphone has a significant effect on the recognition accuracy; thus, researchers generally propose using features invariant to orientation or displacement to achieve this goal. However, those features reduce the capability of the recognition system to differentiate among some specific commuting activities (e.g., bus and subway) that normally involve similar postures. In this work, we recognize those activities by analyzing the vibrations of the vehicle in which the user is traveling. We extract natural vibration features of buses and subways to distinguish between them and address the confusion that can arise because the activities are both static in terms of user movement. We use the gyroscope to fix the accelerometer to the direction of gravity to achieve an orientation-free use of the sensor. We also propose a correction algorithm to increase the accuracy when used in free living conditions and a battery saving algorithm to consume less power without reducing performance. Our experimental results show that the proposed system can adequately recognize each activity, yielding better accuracy in the detection of bus and subway activities than existing methods.

## 1. Introduction

The goal of activity recognition (AR) is to recognize common human activities in real-life settings [1]. AR modules are based on data acquired from sensors such as an accelerometer, video, and GPS. Research on AR began in the 1980s and has become a core technology in the field of healthcare, life care, and wellness, which all require information about users’ life patterns to provide personalized services. Many studies have suggested methods to recognize numerous activities with high accuracy [2,3,4,5,6]. In particular, accelerometers are a popular way to recognize users’ activity. That line of research started by assuming that intrinsic energy would vary with motion, and thus uses accelerometers to measure those variations. Previous research tried to measure the change of motion precisely, so researchers attached many accelerometers to each part of the body and analyzed the resulting data [7,8,9,10,11].

AR methods building upon multiple sensors became less frequent after the introduction of smartphones. The methods devised from previous research required users to buy sensors of different types and attach them to different parts of their bodies. Each sensor is expensive, and the resulting systems are cumbersome to use in real life. However, smartphones are equipped with multi-modal sensors, and many people worldwide (and more people every year) carry one [12]. Therefore, AR research has significantly shifted direction in recent years, and now focuses more predominantly on the use of smartphones. Most AR research on smartphones uses the accelerometer and shows considerably high accuracy in recognizing ambulatory activities, such as staying, walking, and jogging [13,14,15,16,17]. However, it shows low accuracy in recognizing when a user is in a bus or subway [18,19,20]. That discrepancy in accuracy occurs because different physical activities involve certain significant physical differences that are indicated in the magnitude and pattern of acceleration signals. When people are inside a vehicle like a bus or subway, they either stand or sit, relatively motionless. However, it is quite difficult to distinguish between standing/sitting on a static floor or being in a vehicle; no large difference appears in the patterns of rare acceleration signals. This will be discussed further in Section 3.1.

In this paper, we propose movement activity recognition while commuting using accelerometer and GPS data from a smartphone to recognize when a user is staying, walking, jogging, and in a bus or subway train. We do not distinguish between standing and sitting but combined them into staying because it is difficult to distinguish these two activities using only one device. For instance, when we use a smartphone while staying still, the acceleration signal will not show much variation, only the small movements involved in handling the phone. We cannot determine whether the current physical activity is standing or sitting because we do not consider the orientation and position, only the magnitude of the variation. We recognize these five activities (staying, walking, jogging, riding in a bus, and riding in a subway) as basic activities in daily life that occur frequently and can thus be used as source data for a life-log. We use the accelerometer and gyroscope embedded in a smartphone to correct the acceleration data and obtain a fixed signal vector irrespective of the direction of the X, Y, and Z axes. We collected data and extracted features by carrying a smartphone in shirt and pants pockets, in a bag, and in the hand, which are the general positions in which users carry smartphones. We have proposed a correction algorithm to achieve higher accuracy. The purpose of the correction algorithm is to maintain the current activity result unless it is truly changed. For example, if the recognized activity shows walking and there was a misrecognition as jogging in the middle of walking, it compares the current recognized activity and previously recognized activity to conclude whether the final correct activity is walking or jogging. A battery-saving algorithm is also proposed in this paper. According to commute time statistics among OECD countries in 2016 [21], the average commute time takes no more than one hour, which means it will take about two hours a day for commuting considering turnaround time. According to our experiment, when the accelerometer and GPS is turned on all the time, the battery is drained within three hours. Applying the battery-saving algorithm will increase the battery depletion time up to five hours when the sensors are turned on continuously. Our purpose is to let the phone last for a day without recharging. As the application is intended to be used only in the commuting time, it is possible for the phone to last for a day. The contributions of this paper are:

(1) Movement activity recognition with a smartphone without considering position or orientation. We use the Z-axis to extract natural vibration features from a bus and subway and use that information to distinguish staying, riding in a bus, and riding in a subway.
(2) Distinguishing bus riding from subway riding by extracting natural vibration features. Most research is conducted in a constrained environment and thus shows poor results when used in free living conditions.
(3) Correction and battery saving algorithm to increase accuracy and maintain longer battery usage in free living conditions. We propose a correction algorithm to increase accuracy by determining a user’s current activity by referring to previously recognized activity. We also propose a battery-saving algorithm to consume less power while maintaining recognition accuracy.

The remainder of this paper is structured as follows. Section 2 describes related AR works. Section 3 describes our proposed methodology to recognize activities, along with the correction and battery saving algorithms. Section 4 describes our experimental environment and results. Section 5 discusses some issues, and we present our final conclusion in Section 6.

## 2. Related Works

Research on AR using inertial sensors has long been undertaken, with various accomplishments. AR was initially achieved by attaching individual sensors to various body parts. The most remarkable research was conducted by Bao et al. [7]. They attached five 2D accelerometers on the forearm, wrist, pelvis, knee, and calf and recognized activities such as walking, running, standing, sitting, watching TV, cycling, eating, and reading. They showed an average of 84% accuracy independent of user. Banos et al. [22] dealt with sensor displacement problems, which is also a classic problem in the area of inertial sensor-based AR. They also proposed multi-sensor fusion-based asymmetric decision weighting method for robust activity recognition [23].

The challenges for smartphone-based AR are (1) determining whether to perform the processing in the phone or transmit the data to a server, (2) deciding what kind of smartphone sensors to use, (3) handling the position and orientation problem, and (4) recognizing complex activities rather than basic ambulatory activities. At the early stage of the research, the phone served merely as a data collection device [4]. The data were either transmitted directly to the server through online communication or connected to the server after data collection was complete. In both cases, the server performed the whole recognition process. As smartphone performance increased, the recognition process moved to the smartphone itself.

Most AR research uses only an accelerometer, but some studies have used other inertial sensors. For example, Shoaib et al. used an accelerometer, gyroscope, and magnetometer to evaluate recognition results using different combinations of sensors [24]. The accelerometer and gyroscope showed reasonable results even when used independently, but the magnetometer was not sufficient to use alone because of the device orientation problem. Khan et al. used a pressure sensor and microphone along with an accelerometer [25]. They used the microphone based on other research [20,26]. The pressure sensor was used to track altitude—particularly the relative altitude between different points, which could be helpful in recognizing activities that result in an increase or decrease in altitude, such as climbing or descending stairs.

Some researchers tried to achieve position- or orientation-independent recognition. Anjum et al. collected data with the phone in different orientations [27]. To solve the orientation problem, they rotated the three orthogonal reference axes to align with the three transformed axes with eigenvector corresponding to the axes in descending order of signal variation. Henpraserttae et al. proposed a projection-based method for device coordinate estimation to handle the device orientation problem [28]. They transformed all input signals into a single global reference coordinate system. Lu et al. used orientation-independent features, one-time device calibration (which can be perceived by the user), and classification techniques in which activities are modeled by splitting them into several sub-classes, each of which is associated with particular body positions [29].

Some of the research tried to identify vehicles, such as car, bus, or subway. They were to recognize more contexts including vehicles with a single phone with sensors in it. Wang et al. tried to recognize walking, cycling, and riding in a vehicle [18]. They used signal vector magnitude (accelerometer magnitude) to combine three axes of acceleration signals into one to try to recognize activity independent of the orientation of the smartphone, and then extracted vertical and horizontal features from that signal. Bus and subway showed low accuracy compared to the others because of their apparent similarity. The confusion between bus and subway seemed to be caused by extracting vertical and horizontal signals from the accelerometer magnitude signal. In other words, it is difficult to retrieve the original intact signal after the acceleration signal has been combined. Siirtola et al. also worked to recognize the activity of driving a car [19]. They used calibration- and orientation-independent features to achieve device-independent AR. They collected data from five different body locations to achieve position independence, and were able to distinguish activities into two main categories: active (running, walking, cycling) and inactive (sitting, standing, on a table, in a car). Driving a car was more poorly recognized than the others. Han et al. used additional sensors in the smartphone with a hierarchical structure [20]. They recognized staying, walking, running, and shaking using the accelerometer. Shaking indicates either a bus or a subway, and they used the microphone to distinguish between them.

Other research proposed energy-saving methods. Anguita et al. proposed the novel multi-class hardware-friendly support vector machine (MC-HF-SVM) approach, which uses fixed-point arithmetic to recognize activities instead of the conventionally used floating-point arithmetic algorithms to balance the trade-off between recognition accuracy and computational cost [30]. MC-HF-SVM can vary the fixed-point number representation (number of bits) to control accuracy and complexity, leading to improvements in both recognition accuracy and power requirements. Yan et al. tried to save energy by continually tracking the current/ongoing activity of the user and dynamically adjusting sampling frequency and classification features to maintain the optimal value of a specific activity [31]. Morillo et al. compared a series of smartphones and sensors with regard to battery consumption [32]. They then adopted a dynamic sampling rate and duty cycle to reduce the overall battery usage. Liang et al. [33] used a hierarchical recognition method. In order to reduce the computational workload in the feature extraction stage, they first used basic time domain features to measure similarities. Then, they adopted frequency domain feature extraction according to similarities. They also changed the sliding window size dynamically.

## 3. System Design

**Figure 1:**
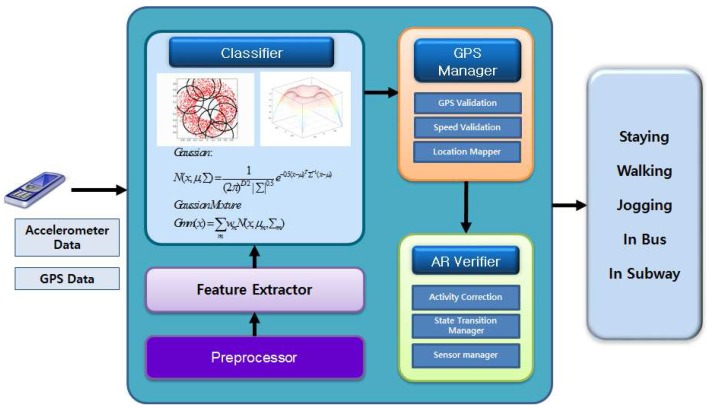
Overall architecture of the proposed methodology. AR: activity recognition.

In this section, we introduce our methodology to recognize staying, walking, jogging, riding a bus, and riding a subway in real-life situations by using a single smartphone without considering any specific placement or orientation. For analysis of the problems, we have used the data collected by ourselves; details are shown in Section 4.2. Figure 1 shows the overall architecture of our proposed method. We collect accelerometer and GPS data from the smartphone. The preprocessor works to combine acceleration signals and remove the gravity. The feature extractor extracts features of time domain, frequency domain, and linear predictive coding (LPC) features. We use a Gaussian mixture model (GMM) classifier for classification. A GPS manager validates the proper GPS signal reception and maps the beacon location. An AR verifier manages the sensor for battery saving and corrects the activity to determine the final activity.

### 3.1. Accelerometer Data Preprocessing

**Figure 2:**
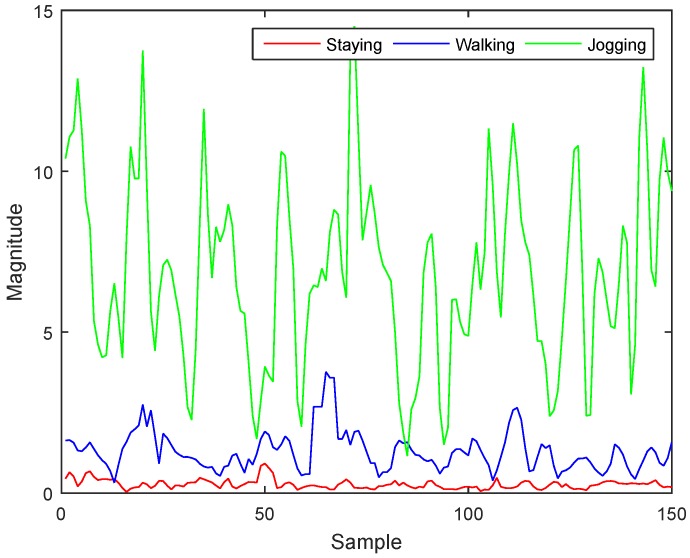
Comparison of staying, walking, and jogging using accelerometer magnitude signals.

Before the emergence of smartphones, AR researchers attached individual accelerometers to the body and extracted features based on the axis with the pattern most characteristic of an activity. However, this method is not applicable for smartphones because people do not carry their smartphones in a fixed position and do not consider the orientation. Thus, we use signal vector magnitude (accelerometer magnitude) to offset the direction information which combines three acceleration signals into one, as shown in Equation (1). If we extract features from the accelerometer magnitude signal, we can easily distinguish staying, walking, and jogging, which all show large differences in pattern and magnitude. Figure 2 illustrates this distinction using our collected data.

(1) $$SignalVectorMagnitude=\sqrt{acc_{x_{i}}^{2}+acc_{y_{i}}^{2}+acc_{z_{i}}^{2}}$$

**Figure 3:**
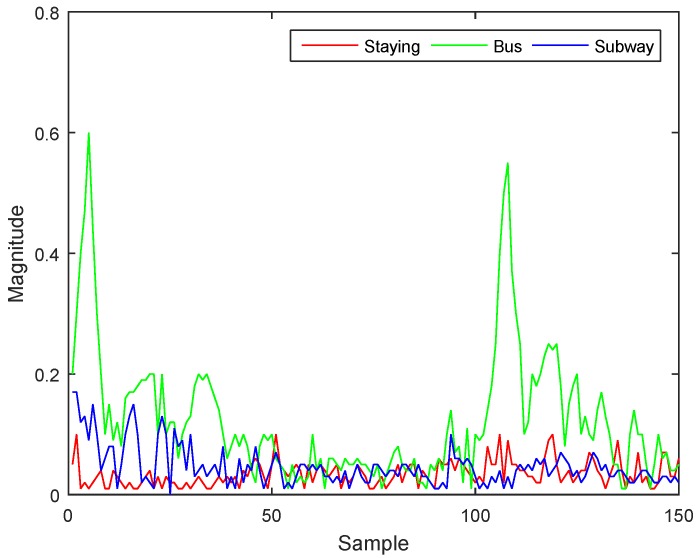
Comparison of staying, riding a bus, and riding a subway using accelerometer magnitude signals.

Although using accelerometer magnitude can distinguish staying, walking, and jogging well, it is hard to use this method to distinguish staying, riding a bus, and riding a subway (as shown in Figure 3). People usually stay still inside the vehicle, which means there is no large difference between staying and riding in a vehicle, and it is also hard to distinguish between bus and subway, as only a small amount of variation occurs while inside the vehicles. Our hypothesis is that each vehicle has a natural vibration that can be used to accurately determine the corresponding activity taking place. For this, we need to take a look into the gravity axis. Existing research used accelerometer magnitude for cases in which the direction of a smartphone was not fixed, because accelerometer magnitude offsets the effect of the axis. However, this method cannot reflect the natural features that occur in each axis. A method was proposed to solve that problem by extracting vertical and horizontal features from an accelerometer magnitude signal [18]. However, that method could not perfectly restore the original characteristics, which degraded the accuracy of vehicle recognition. Therefore, we use a method to fix the acceleration signal using a gyroscope. Thanks to the smartphone environment we are using, android API provides this function, getRotationMatrixFromVector [34,35]. In this way, all three axes of the accelerometer show zero value while placing still in any direction. This will lead us to gain the magnitude of gravity axis (Z-axis) when the phone is moved in up-and-down direction.

### 3.2. Feature Extraction and Classification

**Figure 4:**
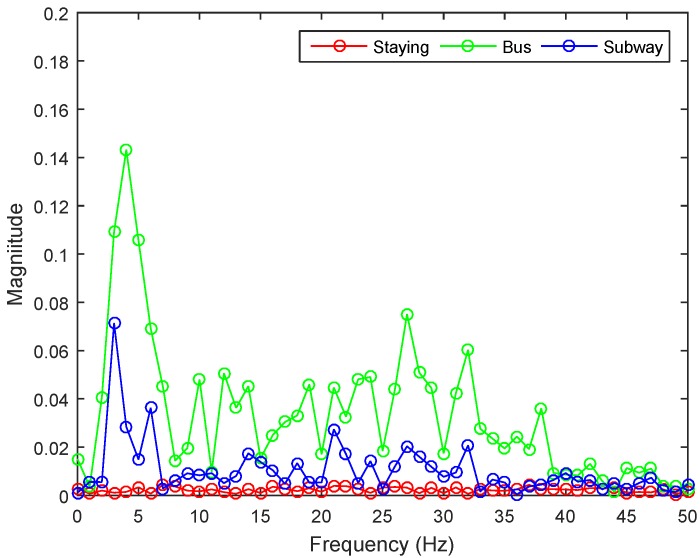
Z-axis signal in the frequency domain for staying, riding a bus, and riding a subway.

For features we use to classify staying, walking, and jogging, we used 10 features from the accelerometer magnitude signal, including the fifth degree linear predictive coding (LPC), one LPC estimation error, three one-second standard deviations (STD), and one mean crossing rate (MCR). These features are used in our previous work, and were already chosen to be best [20]. For classifying two vehicles, we use different features. As mentioned in Section 3.1, our hypothesis is that each vehicle will have its own natural vibration, and vehicles with similar characteristics (such as a car and a bus) will have similar natural vibrations. While inside the vehicles, people will be motionless and the smartphone with only the gravity axis will show large vibration. To observe the differences, we used a fast Fourier transform (FFT) to change the time domain signal into a frequency domain signal to consider a vehicle’s natural vibration frequency. Figure 4 is a representative figure showing the signals for staying, riding a bus, and riding a subway in the frequency domain of our collected data.

The highest frequency shown in the bus is around 5.3 Hz, and is distributed between 3 and 7 Hz. This seems to happen because of variance in the speed of the bus and road conditions. Another characteristic is that the bus shows higher magnitude than staying and the subway. The highest frequency in the subway is around 2.3 Hz, and is distributed between 2 and 4 Hz. Another characteristic is that the subway shows a magnitude similar to staying except between 2 and 4 Hz. In staying, no particular frequency shows high magnitude; thus, staying does not appear to have a natural vibration, unlike the bus and subway. Based on this analysis, we use five kinds of features: mean and standard deviation for staying, bus, and subway; maximum magnitude between 3 and 4 Hz to distinguish bus from subway in the same frequency domain; frequency of maximum magnitude of bus and subway; ratio of 2–4 Hz to the whole frequency; and ratio of 3–7 Hz to the whole frequency. We also extract the same features on the X and Y axes. Even though the Z-axis reflects the natural vibration, the magnitude of the signal is very weak and could easily be contaminated with noise. Therefore, we enhance our accuracy by considering signal changes such as acceleration of the vehicle. For this, we do not need to consider the direction of the vehicle, only the variation in magnitude. Thus, we combine the X and Y signals and transform the result with FFT. We also use features of correlation between the XY and Z axes in both the time and frequency domains to determine the relation between the XY axis and the natural vibration from the Z axis.

There might be unnecessary features among what we have chosen. Therefore, we apply our own feature selection method which measures the feature based on the relevancy and the redundancy from the mutual information of the feature [36]. The relevancy is calculated as in Equation (2):

(2) $$Rel\left(X\right)=\frac{I(C;X)}{log_{2}\left(\left|\Omega_{c}\right|\right)}$$

where *X* is a feature variable, *C* is a class variable, and $\Omega_{c}$ is the state space of *C*. I(C;X) is the mutual information between *C* and *X*, which can be calculated by:

(3) $$I\left(C;X\right)=\sum_{c\in \Omega_{c}}\sum_{x\in \Omega_{X}}p\left(c,x\right)\log_{2}\left(\frac{p(c,x)}{p(c)p(x)}\right)$$

where $\Omega_{X}$ is the state space of the variable *X*; p(c,x), p(c), and p(x) are, respectively, the joint and marginal probability distributions. The redundancy is calculated as in Equation (4):

(4) $$Red\left(X,Y\right)=\frac{I(X;Y)}{log_{2}\left(\left|\Omega_{X}\right|\right)}$$

The mutual information can be calculated by summing over the state space of the variable where the variables should be discretized first. The discretization algorithm is illustrated in Algorithm 1. Once the relevance and the redundancy have been calculated, we utilize the well-known searching mechanism called greedy forwarding to extend the selection of features. The whole selection process is illustrated in Algorithm 2.

After extracting and selecting features, let us assume that ($X^{c}$ is a training data matrix N×K for class *C*, where each row is a training sample, and each column is a feature value. We have chosen a Gaussian Mixture Model for classification as it is suitable to use a mixture model to represent multiple distributions of collected data for multiple dimensions of features [20]. We determine the parametric probability density function of each class, denoted by $p(X^{c}|\lambda^{c})$, where $\lambda^{c}$ is the parameter set that includes the mixing weights and individual Gaussian mean vectors and covariance matrices:

(5) $$p\left(X^{c}|\lambda^{c}\right)=\sum_{i=1}^{M}\omega_{i}N\left(X^{c}|\mu_{i}\sum_{i}\right)$$

where *N* is a Gaussian distribution and is given by:

(6) $$N\left(x|\mu_{i}\sum_{i}\right)=\frac{1}{{\left(2\pi \right)}^{D/2}|\sum_{i}{|}^{1/2}}\exp\left\{-\frac{1}{2}{\left(x-\mu_{i}\right)}^{\prime }{\sum_{i}}^{-1}\left(x-\mu_{i}\right)\right\}$$

The mixing weights must satisfy the following condition:

(7) $$\sum_{i=1}^{M}\omega_{i}=1$$

During the training phase, the parameters $\lambda^{c}=\left(\omega ,\mu ,\Sigma \right)$ are determined to maximize the training data likelihood $p(X^{c}|\lambda^{c})$. In the inference phase, given all the class parameter sets $\lambda^{C1},\lambda^{C2},...,\lambda^{Cm}$ and an input vector *x*, the class label is determined by:

(8) $$c={\mathrm{argmax}}_{c}\left(p\left(x|\lambda^{c}\right)\right)$$

**Algorithm 1:** Feature Quantization
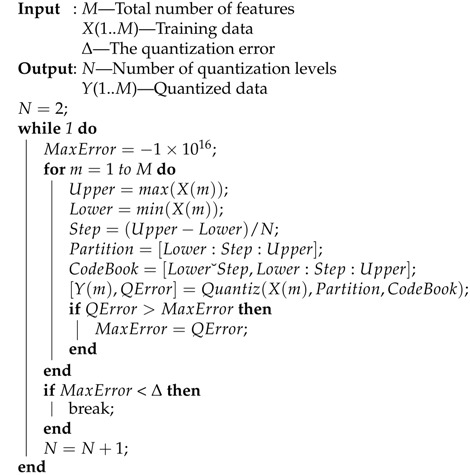


**Algorithm 2:** Feature Selection with Greedy Forward Searching
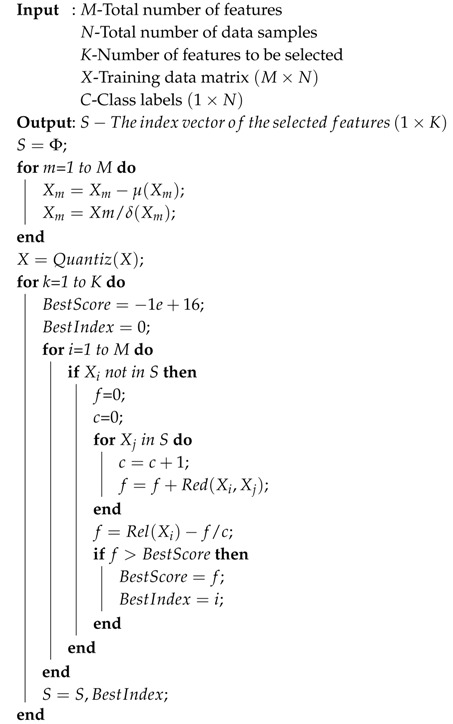


### 3.3. Adding GPS Data

**Figure 5:**
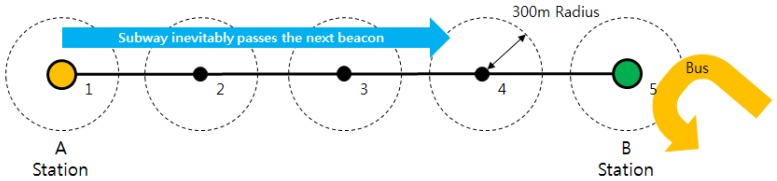
Distinguishing bus and subway on ground level using GPS location data.

So far, this study has focused on acceleration data for activity recognition. If we use GPS outdoors, we can distinguish buses from subways more easily. Therefore, we collect GPS data along with acceleration data to acquire location (latitude and longitude) and speed information. The acquired acceleration data are used for feature extraction and activity recognition, as described in Section 3.1 and Section 3.2. GPS data are used for correction to recognize buses and subways. The accuracy of GPS varies with surroundings and location. If the GPS signal is not received properly, both location and speed data will be incorrect. If the location data are significantly incorrect, the speed could appear higher than is accurate to compensate for the differences between accurate and inaccurate location points. This will cause a problem if the user is actually staying, but the recognized result shows vehicle because of the speed. To prevent that problem, we determine a threshold speed to distinguish staying from buses and subways. The basic idea is that staying will show zero speed, while in reality, this might not be true. We placed three smartphones outside on flat ground for 3 hours while recording latitude and longitude. When we analyzed the latitude and longitude values, they did not maintain linearity; some values showed unnaturalness, which means the value showed significant difference once in a series of similar values. This happened for all three phones, and more than five times respectively. Given that the formula for obtaining speed is distance divided by time, we averaged all of the values of differences and obtained 9.6 km/h. Therefore, we set the threshold speed as 9.6 km/h to differentiate ambulatory activities and vehicle activities. In other words, if the speed from GPS is greater than 9.6 km/h, we assume that the current activity is not staying, walking, or jogging, but either bus or subway. We also use GPS for ground-level sections of the subway. To distinguish between bus and subway on similar courses, we collected five beacons between two stations. Our experiments were conducted in Seoul, South Korea. We input the GPS address of every beacon for the subway stations using Google Maps. To use this method in a different context, the GPS address should be changed. In Korea, the time from one station to the next takes 2–3 min on average. Therefore, we set the time interval to 30 s among the beacons. Each beacon has its own latitude and longitude. If the subway passes from one beacon to another within 30 s, we determine that the user is on the subway. If the user does not pass from one beacon to another within 30 s or passes only one beacon, we determine that the user is on the bus. An illustration of this procedure is given in Figure 5.

### 3.4. Correction Algorithm for Activity Recognition

**Figure 6:**
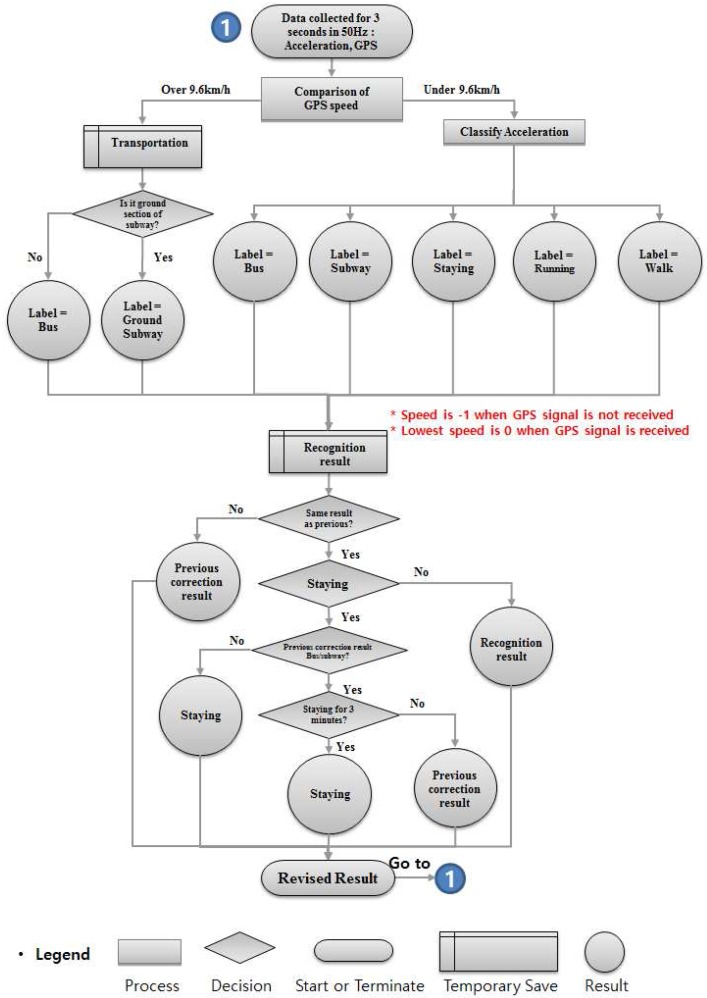
Flowchart of the proposed AR algorithm.

Guaranteeing accurate recognition requires a correction mechanism. During any activity, the user might handle the phone in a way that will change the pattern of acceleration data and greatly affect the accuracy. Bus and subway recognition will also be affected if the GPS signal is not received properly or when the bus or subway train stops at a station. All of these occurrences constitute noise and could lead to incorrect results. To correct this problem, we make the final activity decision only if two sequential results (current and previous) are the same. If they are not the same, we maintain the activity as the last recognized activity. Figure 6 shows the flowchart for this process.

After the data is collected, we consider the speed first. A low speed could be any activity, but a speed greater than 9.6 km/h can only be the bus or subway. The first five data points (15 s) are an initializing stage. Therefore, the first recognized result will be shown at the 15th second. In this first recognition cycle, there will be no correction result in the previous stage, so the correction result will be the same as the recognized result. The correction flow starts from the next cycle. If the recognized result differs from the previously recognized result, the final result will show the same one as previously corrected result, and the current corrected result will be set as the same activity and terminate the flow. If the recognized result is same as the previously recognized result and it is not staying, then the final result is shown as the current activity. Then, it saves the current activity to correction result and terminates the flow. On the other hand, if the recognized activity is the same as the previously recognized activity and both are staying, we take a look into the previously corrected result. If the previously corrected result was not bus or subway, the current activity will be staying. However, if the previously corrected result was bus or subway, we take a look whether the previously recognized results maintained as staying for 3 min. Additionally, if the staying showed for 3 min, the final activity is concluded as staying. If the staying did not show 3 min of staying continuously, the activity will be concluded as the previously corrected result, which will be either bus or subway. The 3 min interval is set to prevent misrecognition as staying. When a bus and subway run on smooth road and rail, the recognition result will frequently show staying. When either vehicle is stopped at a station, it will also show staying due to the lack of movement. So, once the algorithm recognizes the activity as vehicle, and staying occurs in the middle, we disregard this result and force the algorithm to show the result as vehicle. However, we need a breakpoint or the activity will not be changed from vehicle. As mentioned above, the time from one station to another in Korea is 2–3 min for either bus and subway, and the longest traffic light standby time is about 3 min for the bus. So, we set the break time to 3 min. If the staying shows 3 min, now the algorithm determines that the user is actually staying and escapes from the recognition as vehicle.

**Figure 7:**
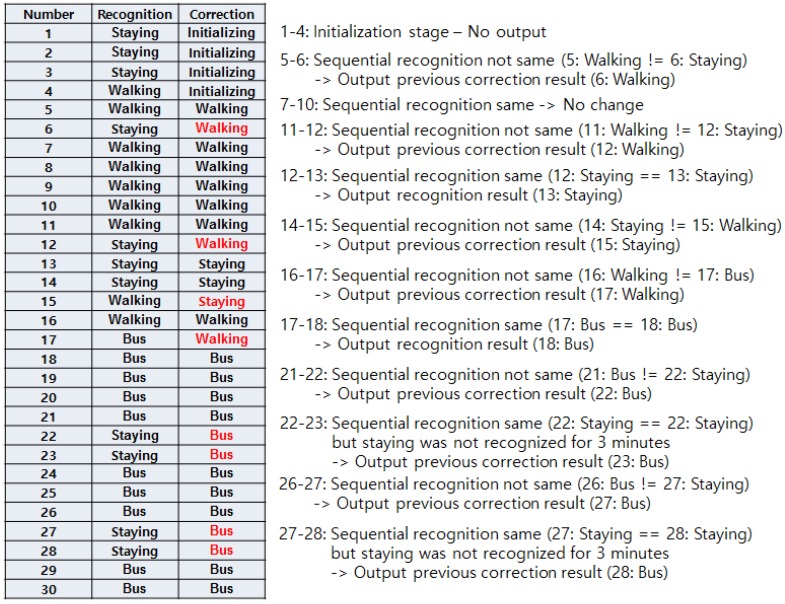
Example of the correction algorithm.

Figure 7 shows an example operation of the correction algorithm. A total of 30 activities were recognized, and the correction was performed for each activity. Note that one activity label is concluded after 3 s and that we set the size of the window in this way. The first four correction results (12 s) are not shown because they are in initialization stage. The fifth result shows the same in both recognition and correction parts as there are no data to refer to from the previous. On the sixth result, the actual recognition changed from staying to walking. However, the previous correction result was walking, so the algorithm disregarded the recognition result and set the correction result as walking. This happened again on the 12th. On the other hand, the 13th recognition result was staying, which is the same as the previous (12th) recognition result. Although the correction result is different from walking, this is regarded as the ground truth activity and the correction result is changed to staying. Taking a look at the 23rd result, the recognition results of both 22nd and 23rd are staying. It seems that the correction result should be changed to staying even if the previous (22nd) correction result was bus. However, as mentioned above, we set a 3 min interval in the vehicle activity. So, the algorithm ignores the recognition result (staying) and concludes the correction result as bus.

**Figure 8:**
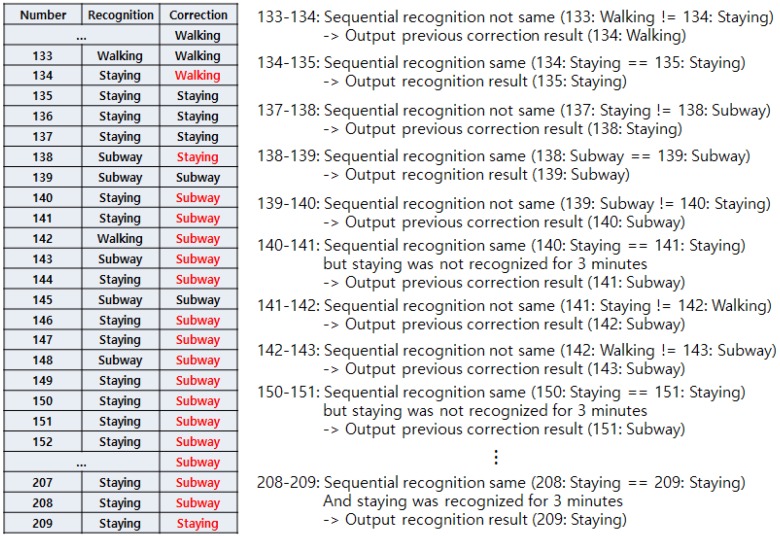
Example of the correction algorithm.

Figure 8 shows another example of the correction algorithm’s operation. Until the 140th result, it is similar to the previous example. After that, if the correction result changes to subway, it ignores the recognition result but keeps the correction result as subway. From the 149th, the recognition result turned to staying until the end. This means that the user actually got off the subway and was staying still. However, due to the 3-min interval problem, the correction result kept showing subway. After 3 min (which means 60 blocks after), the algorithm finally concluded the ground truth activity as staying. This case is the only disadvantage of our algorithm; however, we cannot eliminate this 3 min interval feature because it will lead to substantial error without it.

**Figure 9:**
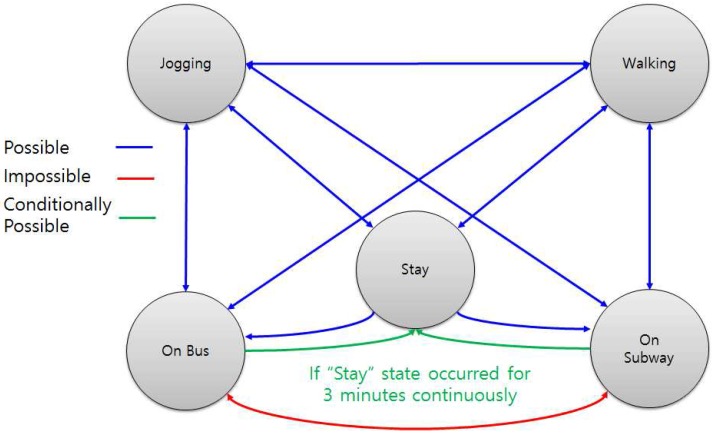
Transition state diagram between activities.

Figure 9 shows the state transition diagram. Staying, walking, and jogging can alternate, but bus and subway cannot be changed to each other due to the nature of reality. Normally, bus and subway cannot be changed to staying, except when staying was misrecognized as bus or subway for 3 min, as shown in the above example.

### 3.5. Battery-Saving Algorithm

**Figure 10:**
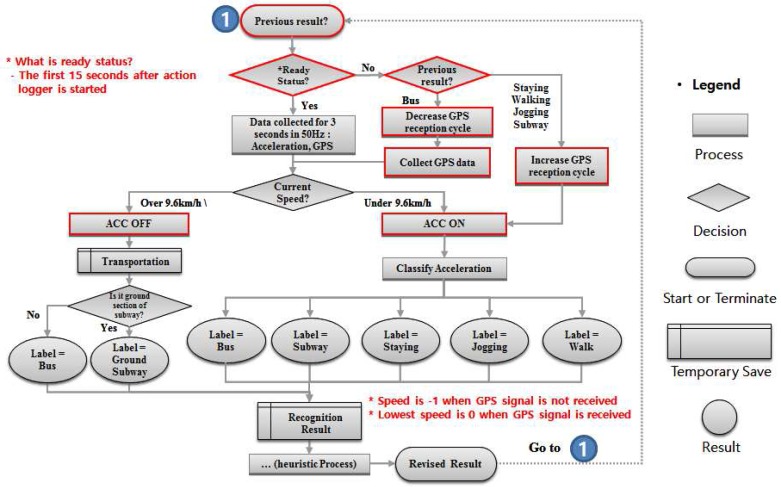
Flowchart of the proposed battery-saving algorithm. ACC: accelerometer.

The method just explained constantly receives GPS and acceleration signals even when data from one sensor does not affect the recognition; this consumes unnecessary battery. To check for differences in sensor battery consumption, we used two phones and fully charged them before each experiment. We created a simple application that uses the accelerometer and GPS and repeated each experiment 10 times. The phone consumes an average of 2% of the battery for 2 h in idle state. However, it consumes 5% of the battery when it runs the accelerometer and 11% when concurrently using GPS. We therefore composed a battery-saving method to prevent rapid battery depletion and guarantee that our recognizer can be used for at least one day. Our algorithm dynamically turns the sensors on and off or changes the sampling frequency according to the activity. If the previous activity was bus and the current speed is more than 9.6 km/h, the recognizer turns off the accelerometer until the speed goes below 9.6 km/h. If the activity is recognized as staying, walking, jogging, or subway, GPS does not affect the recognition, so we extend the cycle for receiving a GPS signal from 1 s to 30 s. Figure 10 shows the battery-saving algorithm adjustment compared to the original algorithm.

## 4. Experiment

In this section, we introduce our experimental environment. We describe the devices used, data collection environment, performance evaluation method, and results. We also describe an experiment conducted in the field.

### 4.1. Experimental Environment

We used a Samsung Galaxy S3 (SHW-E210K, SHW-M440S, GT-I9300T) for data collection and testing. One window contains 150 samples of acceleration data collected for 3 s on the X, Y, and Z axes at 50 Hz. We use the highest speed acquired during 3 s of GPS data. Detailed specifications for the device used in this research are shown in Table 1.

### 4.2. Data Collection Environment

**Figure 11:**
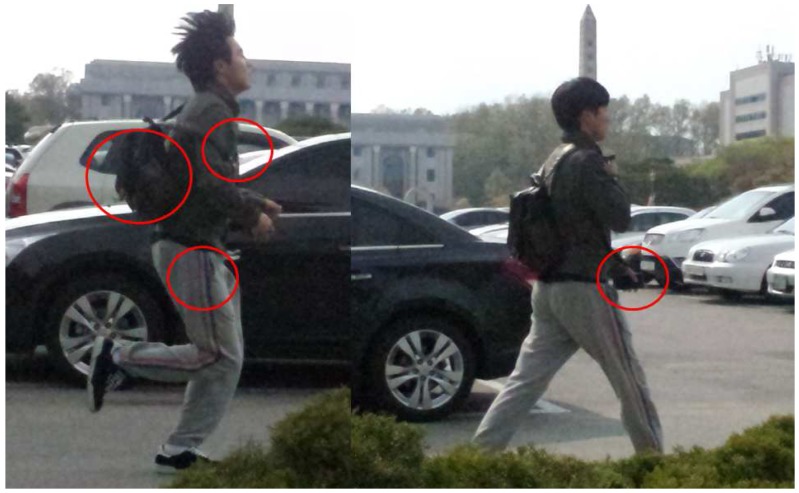
Smartphone carried in four different positions.

We used four smartphones positioned in a shirt pocket, a pants pocket, a bag, and held in a hand for simultaneous data collection, as shown in Figure 11. Those four positions represent common ways people carry their smartphones.

Ten subjects participated in data collection: two females and eight males between the ages of 24 and 28 years. We collected data while staying, walking, jogging, and riding in a bus and subway while carrying the smartphones. We collected both standing and sitting data while on a bus and a subway and while staying. We also used the smartphones while staying (e.g., browsing the internet) in order to prevent misrecognition of the activity because the phone was in use. The total length of datasets collected is shown in Table 2.

Collected data are distributed as shown in Table 3 for performance evaluation.

### 4.3. Performance Evaluation Result

In this section, we show the recognition results based on the accelerometer, along with the results of our proposed method. First, we performed an experiment using an existing method by processing the sensor data with accelerometer magnitude. Based on that, we extracted 10 features: the fifth degree LPC, LPC error, three STD, and one MCR. Table 4 shows the confusion matrix of the result.

Our proposed method additionally uses natural vibration features extracted from the XY-axis and Z-axis and correlations of the XY-axis and Z-axis, for a total of 44 features. Table 5 shows the confusion matrix of the result.

The accuracy for bus and subway was 92.14% and 86.19%, respectively, using accelerometer magnitude—higher than that for our proposed method, which was 89.12% and 81.03%, respectively. However, the existing method had only 77.13% accuracy for staying, whereas our method was 94.10% accurate for the dominant activity in real life. This is a critical issue for any AR system to be used in the real field. Although our method showed lower accuracy in recognizing bus and subway, the confusion between them was only 1.2%, which is very low. Our method showed some delay in recognizing bus and subway, but it did not misrecognize staying as riding in a vehicle. The delay in recognizing a vehicle is fairly short, which might not be a big problem because it takes 2–3 min to move from one station to another.

### 4.4. Experiment for Battery Consumption

**Figure 12:**
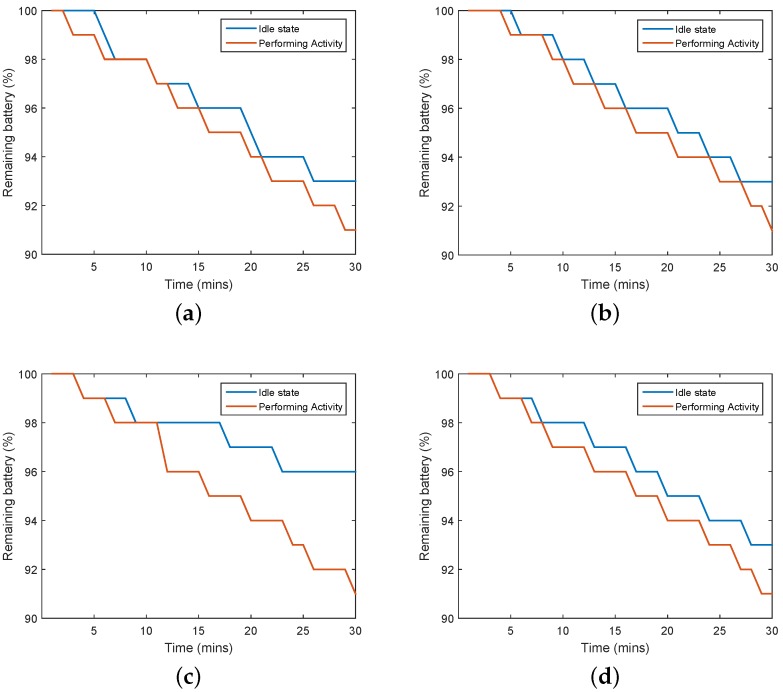
Comparison of the performance of the battery-saving algorithm during different activities. (**a**) Staying; (**b**) Walking; (**c**) Riding in a bus; and (**d**) Riding in a subway.

In this section, we show the results of our proposed battery-saving algorithm. We performed this experiment in the field. Figure 12 shows that our battery saving algorithm conserved energy compared with the normal way. On the bus, battery usage decreased by about 5%—from 9% to 4%—over 35 min, which means the efficiency increased by up to 55%. The bus spends more time running than stopping at bus stops or for traffic, and that turns off the acceleration data. The other activities saved about 2% of the battery from 9% to 7% over 30 min, which means the efficiency increased by up to 22%. Applying the battery-saving algorithm did not affect the recognition accuracy because we applied the correction algorithm.

### 4.5. Experiment in a Free Living Condition

To determine the accuracy of our proposed method, we conducted an experiment in a free living condition, applying both the correction and battery-saving algorithms. We chose five subjects who had not been data collectors in earlier experiments and asked them to carry the smartphone randomly in four different positions. They were told to perform the test list shown in Table 6 and note the result manually each time. We did not include walking or jogging because the accuracy of those activities is high.

As shown in the table below, our proposed activity recognition technique showed good results in free living conditions. Most of the tests were perfect, except for test numbers 1, 2, 5, and 6. For the misrecognized cases, the meaning of misrecognition applying the correction algorithm is that the activity is misrecognized during the whole activity cycle. For example, test number 5 was to check whether our application can recognize “staying” while the user is on board an escalator. It might misrecognize at the beginning to another activity, but it will soon recognize correctly as staying. It may also suddenly misrecognize to a different activity in the middle of boarding time, but this will be regarded as misrecognition and still the corrected result will show the proper activity, which is staying. Among a total of 48 attempts, three times were misrecognized. This means that for the whole time while on board the escalator, the corrected result did not show staying. It did recognize the activity properly as staying in the middle of boarding occasionally, but due to the correction algorithm, it disregarded this result and kept showing the corrected result, which is actually wrong. This misrecognition situation can be escaped when the user performs a different activity by breaking the correction. Other problems occurred when recognizing between staying and subway. Unlike a bus, a subway has small vibrations, which can cause confusion between staying and subway.

Table 7 shows the accuracy with and without applying the correction. The accuracy is based on the field test conducted in Table 6 as walking and jogging is excluded in this table. Accuracy is computed by calculating the correct result among the actual activity from all cases. While not applying the correction, the subway shows poor result, in that it frequently confuses with staying. On the other hand, bus shows reasonable result because it can get help from the GPS and reduce the error of classifying to other activities. Staying shows the best result, as no such factors affect that activity. When the correction method is applied, the accuracy greatly increases, because the function of the correction method is to maintain the current activity until it is surely changed. It is still not one hundred percent complete for several reasons, such as the lack of GPS signal reception, the maintenance of a false activity if it recognizes the activity incorrectly from the beginning, and the absence of movement for a long time inside of a vehicle (traffic jam, accident, etc.).

## 5. Discussion

In addition to collecting the bus data, we also collected a small amount of data while inside a car. We analyzed the acceleration signal and found that the difference between a bus and a car is small. Applying natural vibration features has largely solved the confusion problem for staying, riding a bus, and riding a subway. Because people usually remain relatively motionless inside a vehicle, it is difficult to distinguish the difference among them with simple features, particularly on a subway. As shown in the experimental results in Section 4.4, staying is more confused with riding a subway than riding a bus. As a bus runs on the road, it faces many obstacles, such as an unpaved road or a speed bump and sudden stops from cars cutting in its path or a person on the road. Those changes produce large variations in acceleration signals, which makes it easier to determine that the current state is not staying. On the other hand, subways run smoothly on the rail and do not typically experience sudden interferences. Although the magnitude of natural vehicle vibration is low and difficult for a human to perceive, the sensors are delicate enough to detect this vibration. However, this is not enough. At the beginning, we started to make our work only to use the accelerometer. The offline test showed quite well, while it still showed many errors in the field test because no constant pattern could be found—especially on the bus. So, we decided to include the GPS even though it consumes a lot of battery. The GPS works as an assistant of the main detector and complements the misrecognition of riding a bus. A problem occurs when a user walks inside a subway. A bus is too short to walk for a long time, but a user can walk from the very front cabin to the very rear cabin of a subway, which would take some time. In that case, our recognizer will recognize the activity as walking even though the user is inside a vehicle. If the user stops walking and stays still again, the recognizer will eventually determine the activity to be subway, but confusion could occur between staying and riding a subway. We use a correction algorithm to overcome the practicality problem by referring to the previous activity to decide whether the current activity is well classified. We showed that this method is applicable in free living conditions, but our recognizer is currently limited to the capital city of Korea for exactness because the GPS addresses of the subway beacons are manually input. If this recognizer is to be used in other countries, it will be necessary to input different beacon addresses. We chose four general positions in which people carry smartphones, but many other positions are possible. We collected data from the trousers front pocket, but people also carry their phones in their hip pockets, inside pockets, and coat pockets. Likewise, we collected data using only backpacks, but people also carry their phones in purses, handbags, and briefcases. As we experimented with those different positions, our algorithm continued to perform well most of the time, but mistakes occurred occasionally. Finally, the number of activities is limited in this work because we wanted to reflect the position and orientation free concept and to maximize practicality. Smartphones are used by most people, so no one needs to buy additional sensors for a daily life activity recognizer. However, this single device can detect only overall body movements, not detailed movements performed by a hand or leg. To extend the recognizable activities, it is necessary to include more sensors or fix the position.

## 6. Conclusion

In this research, we proposed an activity recognition method using an accelerometer and GPS in a smartphone environment to be practical in free living conditions. We separated activities into two categories: ambulatory activities and vehicles. To detect vehicles, we assumed the natural vibrations of two different vehicles and extracted the features. To guarantee practicality, we applied orientation- and position-free concepts, a correction algorithm to increase accuracy in free living conditions, and a battery-saving algorithm with no accuracy degradation. The outcome of our research will greatly help to recognize user activity in daily living. There are some limitations to increasing recognizable activities using a single device with a free carrying environment. In future work, we will add additional activities while complying with minimum sensor device requirements, such as using a smartphone and smartwatch.

## Figures and Tables

**Table 1:** Specifications of the Samsung Galaxy S3.

Smartphone	Galaxy S3
OS	Android 4.1.2 (Jellybean)
CPU	Quad core 1.2 GHz
Memory	1 GHz DDR2
Sensors	Accelerometer, Gyroscope, GPS, etc.
Network	3G, WiFi

**Table 2:** Total length of the collected datasets.

Activity	Bus	Subway	Jogging	Staying	Walking	Total
Number of Samples (s)	26,826	9996	3030	34,212	19,446	93,510

**Table 3:** Distribution of the collected dataset for training, validation, and testing.

Activity	Training (80%)	Testing (20%)	Total
Bus	7153	1789	8942
Subway	2665	667	3332
Jogging	808	202	1010
Staying	9123	2281	11,404
Walking	5185	1297	6482
Total	24,934	6236	31,170

**Table 4:** Confusion matrix for AR using 10 features (Average accuracy: 0.8954).

Activity	Bus	Subway	Jogging	Staying	Walking
Bus	0.9214	0.0414	0.0001	0.0331	0.0040
Subway	0.0192	0.8619	0	0.1168	0.0021
Jogging	0	0	0.9713	0	0.0287
Staying	0.0452	0.1759	0	0.7714	0.0075
Walking	0	0.0004	0	0.0510	0.9486

**Table 5:** Confusion matrix of AR using the proposed method with 44 features (Average accuracy: 0.9182).

Activity	Bus	Subway	Jogging	Staying	Walking
Bus	0.8912	0.0119	0	0.0924	0.0045
Subway	0.0114	0.8103	0	0.1783	0
Jogging	0	0	1	0	0
Staying	0.0100	0.0481	0	0.9411	0.0008
Walking	0	0.0004	0	0.0510	0.9486

**Table 6:** Test list to test the performance of the proposed method in free living conditions.

No.	Test List	Result (Correct Result/Total No. of Times)
1	Standing on a subway	33/34
2	Sitting on a subway	13/14
3	Standing on a bus	14/14
4	Sitting on a bus	33/33
5	Standing on an escalator (to see whether it misrecognizes escalator as a bus or subway)	45/48
6	Turning on the activity recognition application while on a subway	46/48
7	Turning on the activity recognition application while inside a bus (to see whether it misrecognizes the activity while receiving GPS signal)	8/8
8	Seeing whether it turns to a different activity while the bus is stopped for a traffic signal or a bus stop for a long time (2–3 min)	6/6
9	Riding on a bus for more than 2 h (to see whether it misrecognizes the bus as a subway)	14/14
10	Waiting for a bus or subway at the station for a long time (to see whether it misrecognizes the wait as a bus or subway)	52/52
11	Test all activities, including transferring to bus and subway, for at least 2 h	19/19

**Table 7:** Accuracy with and without applying the correction method from the field test.

Activity	Accuracy without Applying Correction	Accuracy Applying Correction
Bus	81	97
Subway	63	95
Staying	93	98
